# Survey of Argentine Health Researchers on the Use of Evidence in Policymaking

**DOI:** 10.1371/journal.pone.0125711

**Published:** 2015-04-30

**Authors:** Adrijana Corluka, Adnan A. Hyder, Elsa Segura, Peter Winch, Robert K. D. McLean

**Affiliations:** 1 Department of International Health, Johns Hopkins Bloomberg School of Public Health, Baltimore, Maryland, United States of America; 2 National Institute of Parasitology “Dr. M. Fatala Chaben”, Ministry of Health, Buenos Aires, Argentina; 3 Policy, Strategy, and Evaluation Division, International Development Research Centre, Ottawa, Canada; 4 Evaluation Unit, Canadian Institutes of Health Research, Ottawa, Canada; Johns Hopkins Bloomberg School of Public Health, UNITED STATES

## Abstract

**Objective:**

In this study, Argentine health researchers were surveyed regarding their perceptions of facilitators and barriers to evidence-based policymaking in Argentina, as well as their publication activities, and research environment satisfaction.

**Methods:**

A self-administered online survey was sent to health researchers in Argentina. The survey questions were based on a preceding qualitative study of Argentine health researchers, as well as the scientific literature.

**Results:**

Of the 647 researchers that were reached, 226 accessed the survey, for a response rate of 34.9%. Over 80% of researchers surveyed had never been involved in or contributed to decision-making, while over 90% of researchers indicated they would like to be involved in the decision-making process. Decision-maker self-interest was perceived to be the driving factor in the development of health and healthcare policies. Research conducted by a research leader was seen to be the most influential factor in influencing health policy, followed by policy relevance of the research. With respect to their occupational environment, researchers rated highest and most favourably the opportunities available to present, discuss and publish research results and their ability to further their education and training. Argentine researchers surveyed demonstrated a strong interest and willingness to contribute their work and expertise to inform Argentine health policy development.

**Conclusion:**

Despite Argentina’s long scientific tradition, there are relatively few institutionalized linkages between health research results and health policymaking. Based on the results of this study, the disconnect between political decision-making and the health research system, coupled with fewer opportunities for formalized or informal researcher/decision-maker interaction, contribute to the challenges in evidence informing health policymaking in Argentina. Improving personal contact and the building of relationships between researchers and policymakers in Argentina will require taking into account researcher perceptions of policymakers, as highlighted in this study.

## Introduction

Despite evidence indicating that policymakers and health professionals consider ‘homegrown’ research to have greater weight than research from other countries [1, 2], and despite researchers being influential in reframing the way health policy issues are seen [3] and identified as factors affecting uptake of their research [4], there has been little empirical research in developing countries on the involvement of health researchers in the policy process [5]. In addition to case studies on disease prevention programs[6] and research capacity strengthening [7], past research focussed on developing country health researchers has included studying researchers’ perceptions of community-based research [8, 9] or their perceptions on ethical review of health research, including institutional review boards (IRB) reviews and informed consent [10]. Lavis et al. (2010) surveyed 308 researchers in 10 low- and middle-income countries (LMICs) on bridging the gap between research, policy and practice, in one of four clinical areas relevant to the Millennium Development Goals [11]. More recently, a survey of health researchers in the Eastern Mediterranean Region (EMR) explored researchers’ views and experiences regarding the role of health systems and policy research evidence in health policymaking in the EMR, including the factors that influence health policymaking, barriers and facilitators to the use of evidence, and the factors that increase researchers' engagement in knowledge, transfer, and exchange [12].

To our knowledge, Lavis et al.’s (2010) and El-Jardali et al.’s (2012) studies are the only other surveys of health researchers in LMICs that have been conducted in the area of knowledge translation. At the time we conducted this research, no published study had explored LMIC country researchers’ perceptions of evidence-based policymaking in their countries, nor what they perceive to be facilitators and barriers to the use of research in decision-making and health policymaking. In the context of national health research systems in South America, a preliminary survey was conducted by Almeida (2001) on systems and services research in Brazil, Argentina, Uruguay and Paraguay to assess the present situation and capacity-building needs [13]. Although challenged by low survey response rates, results indicated that there was a need for clearer theoretical and conceptual delimitations of health systems and services research in Argentina and the other Southern Cone countries. For Latin America in general, and Argentina in particular, there remains a need to develop a greater understanding of the role that health researchers play in research uptake and utilization in policymaking. This research was conducted at a time where Argentine health researchers were believed to be becoming more engaged in policy processes [14], evidenced in part by the creation of the Argentine Forum for Health Research (FISA, in Spanish). FISA’s purpose was to generate an environment for discussion of health research priorities in Argentina [15].

Unlike the study by Lavis et al. (2010), which focused on specific medical and public health interventions, we chose not to focus on a specific health topic for this research. Recognizing the dearth of research on the basic understanding of researchers as critical factors in evidence-informed policymaking [4], we developed three related objectives for this study.

Our first objective was to determine researchers’ perceptions of health policymakers and the policymaking process. Secondly, we wanted to determine what Argentine researchers perceived to be facilitators and barriers to the use of health research in health policymaking. Our final objective was to gain an understanding of the health researcher’s dissemination activities and research working environment in Argentina. Achieving all three objectives would establish a starting point from which to understand the supply side of the research-to-policy spectrum in Argentina and identify opportunities to enhance evidence informing policymaking.

## Methods

### Survey Content

The contents of this survey were purposefully designed to build on the results of the semi-structured in-depth interviews of the two previous phases of this work, which comprised qualitative studies. The first study consisted of interviewing policymakers in five LMIC and one high-income country [16], with a view to better understand their perspectives on the use and impact of research in the health sectors of their respective countries. The second study consisted of interviews with Argentine health researchers regarding their perceptions of the facilitators and barriers to evidence use in health policymaking, what characteristics of health research (such as inclusion of summaries, or, research leadership) they believe influenced research use by policymakers, and perceptions of policymakers’ motivations [17].

Building on the results of these previous studies, including the organizing framework proposed in Corluka et al (2104), several themes emerged that we wished to further investigate. Primarily, we aimed to gather further data on the perspectives of health researchers regarding the knowledge translation process. Secondly, we aimed to collect data that would allow one to better understand health researcher capacities in Argentina, and how health researchers’ work environment might enable and enhance researcher capacity and productivity. Most capacity questions in the survey we implemented were derived from a core set of indicators and descriptive variables developed by the WHO’s Health Research Systems Analysis (HRSA) Initiative [18]. More information on the HRSA Initiative and the variables we included in our survey can be found in S1 File.

### Survey Design

We designed an online survey that was self-administered by Argentine health researchers over seven weeks in May and June 2010. The survey was pilot tested twice in Spanish, with native Spanish-speaker health researchers from South America (including Argentines) and non-native Spanish speakers. The survey was back-translated into English for quality-control purposes. The final Spanish version of the survey was verified by Argentine collaborators for clarity, grammar and spelling. No differences or difficulties were reported in the loading or formatting of the survey, even when using different Web browsers (e.g. Mozilla Firefox versus Internet Explorer) and computer systems (e.g. PC versus Mac). As every researcher contacted in this study had an email address, Internet access by the study population was not an issue.

The Web survey was designed and implemented using the SurveyMonkey program (SurveyMonkey.com, LLC: Palo Alto, California, USA). A list of strategies we used to improve response rate, such as the inclusion of motivational messaging [19], can be found in S2 File.

### Survey Sampling

In order to access the greatest number of Argentine health researchers doing research with applications to population health, a list of email addresses of health researchers was requested from the Argentine National Council of Scientific and Technical Research (CONICET). As part of the Ministry of Science, Technology and Innovation, CONICET directs and co-ordinates most of the scientific and technical research done in public universities and institutes in Argentina. We received two databases of names and email addresses of researchers working in the areas of medical sciences and clinical research, for a total of 649 researchers. Separately, we had compiled a database of the names and email addresses of 52 Argentine health researchers doing public health research; these were drawn from personal contacts, scientific publications and conferences and were not contained in the CONICET databases. After resolving duplicate email addresses, errors and expired email addresses, the combined database was reduced to 647 researcher email addresses. This recruitment approach was somewhat similar to Kho et al.’s (2010) which included identifying potential participants using membership lists from professional associations, known research/clinician collaborations, and professional entities found on the Internet [20]. However, we did not randomly sample from our population. Instead, we approached all researchers whose contact information we had (i.e. a census approach).

To resolve the issue of bounce backs, the secondary and/or tertiary email addresses, if included in the database, were used. If there were no secondary or tertiary email addresses supplied, the literature and Internet were searched for a possible alternative email address for the researcher.

We limited reminders to two emails, as a slight decrease in response rates have been observed among those receiving the largest number of reminders [21]. These reminders were sent at 20 days and 30 days, respectively, after researchers were originally asked to participate in the survey.

### Response Rate Calculation

To calculate the survey response rate, we used a simple response rate calculation [20, 22]. In these calculations, when an error message or bounce back email occurred after sending an email to supposedly accurate and valid email addresses, it was assumed that the respondent did not have access to the survey. These individuals were subtracted from the denominator; 34.9% of researchers accessed the survey and 29.4% completed the survey.

### Statistical Analysis

Statistical analysis was performed using Stata version 9.0 [23]. The significance level was set at alpha = 0.05 (two-tailed). As the variables were not normally distributed, comparisons were assessed using the non-parametric Kruskal-Wallis, Wilcoxon-Mann-Whitney and Fisher’s exact tests. Sex, education and experience differences were tested for all outcome variables analysed. Due to the ordinal nature of the response scales, all correlations are non-parametric Spearman correlations.

### Ethics Statement

This study was approved by the Institutional Review Board of the Johns Hopkins Bloomberg School of Public Health in Baltimore, Maryland, USA as well as by the Ethics Committee of the Hospital Francisco Muniz in Buenos Aires, Argentina. Researchers were emailed an invitation to participate in the survey which contained all the information about the study and served as a consent form. Participants were informed that by clicking on the link, they were providing consent to be in the study. Researchers were not sent a notification email message in advance of being invited to participate in the survey and being sent the link. We did not offer an incentive for participation.

## Results

### Respondent Characteristics

Approximately 56.8% of respondents were female and 80.2% of overall respondents had a PhD, with 44.6% of respondents having more than 20 years of research experience (Table 1). The majority respondents identified themselves primarily as researchers in a federal government-funded institution (69.1%), and more specifically a public university setting (67.4%). Nearly all the researchers surveyed had a doctorate or a medical degree or both, and nearly 90% had more than 10 years of research experience. As was expected given the sampling frame, the majority of respondents worked in laboratory sciences (72.3%), while others categorized their area of research as clinical medicine (23.6%) or pertaining to public health (20%). It should be noted that ‘public health research’ is not a commonly used research classification in Argentina.

**Table 1:** Characteristics of Researcher Respondents.

**Sex**	Female	128 (56.6%)	N = 226
	Male	98 (43.4%)	
**Years of Research**	1–5	5 (2.2%)	N = 224
	6–10	19 (8.5%)	
	11–15	51 (22.8%)	
	16–20	49 (21.9%)	
	21 +	100 (44.6%)	
**Level of Education**	Licentiate*	3 (1.3%)	N = 226
	Masters (e.g. Masters of Public Health)	3 (1.3%)	
	Doctor of Medicine	7 (3.1%)	
	Doctor of Philosophy (PhD)	180 (79.6%)	
	MD & PhD	33 (14.6%)	
**Area of research**	Economics	2 (0.9%)	N = 224
	Sociology	5 (2.2%)	
	Public Health	46 (20.5%)	
	Clinical Medicine	54 (24.1%)	
	Laboratory Sciences	159 (71.0%)	
	Epidemiology	27 (12.1%)	
	Toxicology	14 (6.3%)	
	Environmental Health	5 (2.2%)	
	Mental Health	10 (4.5%)	
	Biostatistics	2 (0.9%)	
**Place of Work**	Public university/government-funded institution	159 (82.4%)	N = 193
	Private university	11 (5.7%)	
	International organization (e.g. United Nations, World Bank)	0 (0%)	
	Non-governmental organizations (e.g. think tank, service provider NGOs)	18 (9.3%)	
	Private company/industry	5 (2.6%)	

*The *Licentiate* degree (Spanish: *Licenciatura*) is a four to six year degree considered equivalent to an M.Sc. or M.A. in North American universities.

### Researcher Activities

Respondents submitted 185 proposals to an IRB in the last year, with 611 proposals submitted in the last five years. On average, 1.16 proposals per survey respondent were submitted to an IRB in the past year, with approximately 3.4 proposals per respondent submitted over the last five years.

The overwhelming majority of respondents (88.6%) disseminated their research via publication in journals, books, reports, etc. while fewer primarily presented their work at conferences (11.4%). No researchers used Web listserves to disseminate their work. Over 91% of respondents indicated that they were able to access national and international health journals (in paper or electronic version).

Argentine researchers surveyed published overwhelmingly in peer-reviewed English journals, with an average of 10.5 articles published in the last five years (from 2005–2009), compared to 3.3 articles in peer-reviewed Spanish-language journals (Table 2).

**Table 2:** Publication and Dissemination Patterns of Argentine Researchers (2005–2009).

Number of:	Response Average	Response Count (N)
Articles published in peer-reviewed journals, in Spanish	3.28	109
Articles published in peer-reviewed journals, in English	10.50	207
Published books	1.39	94
Written/published reports	5.55	80
Working papers	2.98	25
Systematic reviews (e.g. Cochrane reviews or others)	1.80	30

### Researcher Perceptions of Decision-Makers

When asked about their perceptions of research use by decision-makers, the researchers surveyed believed that decision-maker self-interest was the driving factor when decision-makers were formulating health and healthcare policies (Table 3). Decision-makers were perceived to be only “a little” motivated by research results or the health care needs of the population.

**Table 3:** Decision-maker* motivations in health policy development, as perceived by health researchers.

	Not at all motivated	A little motivated	Somewhat motivated	Highly motivated
A. Meeting the healthcare needs of the population	9.6% (19)	**43.7% (86)**	36.5% (72)	10.2% (20)
B. Cost-effectiveness of intervention	14.1% (27)	**37.0% (71)**	26.6% (51)	22.4% (43)
C. Equity and fair distribution of benefits	28.4% (55)	**43.3% (84)**	24.2% (47)	4.1% (8)
D. Poverty alleviation	31.3% (61)	**44.1% (86)**	20.5% (40)	4.1% (8)
E. The impact of policy on health care providers	17.1% (33)	**31.1% (60)**	24.9% (48)	26.9% (52)
F. Results of health research	29.2% (56)	**47.9% (92)**	19.3% (37)	3.6% (7)
G. Cost to the state	5.2% (10)	27.2% (52)	23.0% (44)	**44.5% (85)**
H. Meeting donor requirements	6.8% (13)	25.3% (48)	29.5% (56)	**38.4% (73)**
I. Self-interest	2.1% (4)	10.4% (20)	27.6% (53)	**59.9% (115)**

* where decision-makers are defined as policy advisors, political advisors, politicians.

The majority of researchers surveyed believed that Argentine decision makers are only “a little” aware of the most urgent problems in the country, with over 90% believing that decision makers have little-to-no knowledge with respect to understanding research. Only 3.1% of researchers surveyed indicated that they were involved in health policy and contributed to decision making on a somewhat regular basis, while 15.5% were involved and contributed sometimes. The remaining researchers surveyed (81.4%) had never been involved in or contributed to decision-making. At the same time, 50.8% indicated that they would like to get involved in policymaking processes, but only sometimes. Approximately 11.7% of researchers did not want to be involved in policymaking processes.

There was a statistically significant relationship between researchers’ perceptions of decision-makers’ research knowledge, and researchers’ experiences having contributed to decision-making (p = 0.003, Fisher’s exact test). Five out of the six who had often been involved in policymaking rated decision-makers research knowledge to be poor, while the remaining researcher indicated the decision-makers to be very knowledgeable about research.

There was no statistically significant difference between male and female researchers wanting to be involved in the policymaking process, nor between number of years of research or level of education.

The majority of researchers surveyed (71.9%) believed that decision makers should always be concerned with using research evidence to develop health policies, while 60.7% surveyed believed that decision makers should consult researchers in formulating health policies. In addition, over 93% of researchers surveyed believed that the rules and regulations currently in force in Argentina and governing health systems and reform are “sometimes” or “never” based on research findings.

### Contextual and Environmental Factors Influencing Research-to-Policy

Policymakers’ personal interests, the government budget, and personal contact between researchers and decision-makers were the three most important factors perceived by researchers to influence health research impact on health policy development (Table 4).

**Table 4:** Influence of Contextual and Environmental Factors on Research Use for Policy Development.

	Not at all	A little	Somewhat	Very much
High turnover of staff that make health policy decisions	11.3% (22)	20.1% (39)	27.8% (54)	**40.7% (79)**
The amount of trust between politicians and researchers	9.3% (18)	14.0% (27)	28.5% (55)	**48.2% (93)**
Political instability	5.8% (11)	10.5% (20)	21.5% (41)	**62.3% (119)**
The government budget (national, provincial)	2.1% (4)	7.8% (15)	19.7% (38)	**70.5% (136)**
The personal interests of the political advisor/analyst	2.1% (4)	4.7% (9)	21.1% (40)	**72.1% (137)**
The support/promotion of international health agencies (e.g. PAHO)	5.8% (11)	18.4% (35)	**45.3% (86)**	30.5% (58)
International support	5.2% (10)	24.1% (46)	**40.3% (77)**	30.4% (58)
Lack of information/knowledge about research	3.6% (7)	10.4% (20)	25.0% (48)	**60.9% (117)**
Personal contact between researchers and decision makers	4.2% (8)	5.8% (11)	25.4% (48)	**64.6% (122)**
Pressure from the Argentine public/public opinion	25.9% (49)	**31.7% (60)**	24.3% (46)	18.0% (34)
Support of national organizations	6.9% (13)	28.0% (53)	**41.8% (79)**	23.3% (44)
Attitudes of health professionals to address these issues	3.7% (7)	18.2% (34)	**46.0% (86)**	32.1% (60)
The media/press	5.3% (10)	19.5% (37)	**38.4% (73)**	36.8% (70)
Cost of implementing the research recommendations	4.2% (8)	19.6% (37)	36.5% (69)	**39.7% (75)**
Licensing and availability of pharmaceuticals	3.3% (6)	17.4% (32)	**40.2% (74)**	39.1% (72)

Research was perceived to be most influential in policy development when the research had been conducted by a research leader (Table 5). Policy relevance of the research was also viewed by researchers to influence whether research results would be used in policymaking. Other facilitators and barriers to the use of research in policymaking that were noted by researchers in the optional open-ended responses text box can be found in Table 6, organized by major associated theme (e.g. governance, community/group and political levels).

**Table 5:** Characteristics of Research Facilitating its Use in Decision-Making.

	Not at all	A little	Somewhat	Very much
Research was led by a leader in research	5.4%	13.5%	33.0%	**48.1%**
Research is relevant to specific policies	3.8%	16.2%	38.9%	**41.1%**
Research includes a summary with clear recommendations	5.5%	25.1%	**35.5%**	33.9%
Research confirms current policy/policy recommendations	9.3%	15.3%	**38.3%**	37.2%
Research challenges current policy/policy recommendations	17.2%	**32.2%**	27.2%	23.3%
Inclusion of cost data in the research (e.g. economic evaluations)	8.8%	22.7%	**34.8%**	33.7%
Quality of the research	9.1%	24.6%	**35.3%**	31.0%
Development and use of clinical practice guidelines	8.2%	25.3%	**40.7%**	25.8%
Publications in medical journals	9.6%	30.3%	**36.7%**	23.4%

**Table 6:** Typology of Specific Barriers and Facilitators Named in the Survey.

	Barriers	Facilitators
**Governance Level**	The Ministry of Health is separate from the Ministry of Science and Technology.	Larger budget and better distribution of economic resources.
	Not respecting the provision of resources for research according to priority thematic areas.	The current criteria for granting subsidies for research should be improved.
	Usually, if research is taken into consideration by the decision-makers, it is research conducted by government organizations. A more general and wider viewpoint, such as that by the universities, doesn't seem to interest policymakers.	Use of the State's own intellectual resources to develop health policies such as those residing in institutions like CONICET and the National Agency for the Promotion of Science and Technology.
	The majority of the scientific information is in English due to the dominance of English as the language of science, and the politicians/policymakers are too busy to translate them or consult researchers.	Coordination between health centers and policy makers with research institutes in order to establish priority research topics; requires a budget dedicated to this, and for the process to be monitored.
**Community/ Group Level**	Lack of practicing evidence-based medicine by a large proportion of physicians and the consent of this situation by health authorities.	Increased communication between researchers of proven quality (eg researchers of CONICET) and parliamentarians and health policy developers.
	There is no exchange between research groups in the medical sciences and people linked with public health policy.	Forming committees of health experts (which include researchers) for public health decision-making.
**Political Level**	Lack of political will to improve the area of health and confront power groups which would lose their privileges.	There should be clear and transparent interaction between physicians, researchers and politicians.
	Fundamentally, political interests shift; decision-makers are proud and arbitrary.	Promoting the linking of scientists and those responsible for health policies through meetings and joint projects; the selection of experts should not be by political connection, but by scientific experience.
	Lack of public information about strategies and short-, medium-and long-term health policies.	Cooperative research with specific political actors and/or by province or region.

### Work Environment

The majority of respondents (67.5%) were located and working in the capital of Buenos Aires, while the remaining respondents worked in 13 other provinces.

In rating various characteristics of their working environment, the most favourably rated aspect was that researchers had opportunities to present, discuss and publish research results. Researchers also had opportunities to further their education and training (Table 7). However, salaries were considered to be inadequate and the quality of the work space and research facilities was rated as lacking, both which received the lowest rating. There were no statistically significant differences between females and males regarding organizational/workplace characteristics. The only statistically significant difference appeared in the rating of ‘presence and access to research networks’ linked with years of research experience. Further analysis showed that most inexperienced researchers placed the highest value on this factor, with the second highest value placed by those with more than 20 years experience.

**Table 7:** Researchers’ Ratings of their Research Environment.

Organizational Characteristic	Researchers' Average Rating (out of 100, where 100 is ideal/perfect)	Differences between males and females (p-value*)	Years research experience (p-value**)
There are opportunities to present, discuss and publish your research results.	73	0.2849	0.7852
There is opportunity to continue your education and training.	70	0.4864	0.7328
There is adequate access to published research.	63	0.4814	0.1790
The work environment encourages collaboration with other researchers.	60	0.0620	0.1435
There is a presence of and access to health researcher networks.	57	0.2178	**0.0020**
The funding process is transparent and clear.	56	0.4795	0.4318
The quality of the work space and research facilities is adequate.	48	0.9399	0.2534
There are opportunities for the promotion and nurturing of your career.	48	0.0620	0.4227
The salaries of health researchers are suitable.	40	0.2268	0.3499

*two-tailed T test, alpha = 0.05

**one-way ANOVA.

Researchers surveyed indicated that they would most like to improve the physical research infrastructure and have more current technologies and equipment. There were calls for more and better financing of research supplies and equipment and a need to train more laboratory technicians or junior researchers. There was also a need for transparent funding processes. Geographic inequities in health research project funding were noted, such as inferior working conditions and a lack of funding for the economically disadvantaged provinces compared to the federal capital of Buenos Aires.

## Discussion

This survey was conceived as the last phase of a mixed methods study focussed on Argentine health researchers. Although originally we had intended to focus solely on Argentine health researchers’ perceptions of the facilitators and barriers of research use in health policymaking, during the qualitative research phase where health researchers were interviewed, and which preceded the survey, themes linked to the importance of researchers’ working environments began to emerge [24]. Based on these interviews, it became of interest to us to identify Argentine researchers’ research dissemination activities, and determine which aspects of researchers’ work environments enabled, or became barriers, to researchers contributing to a strengthened national research system. As such, using the opportunity provided by surveying a large number of Argentine health researchers, their perceptions were sought regarding the space in which they worked and produced research, as well as how they perceived research use in Argentine health policymaking.

Argentine researchers’ rating of professional opportunities and access to professional networks, as well as their demands for better quality work space and research facilities, are reflected in other countries. A 2003 case study by the United States Veterans Administration compared centres with high researcher satisfaction to those with low survey satisfaction scores [25]. The researchers found that highly satisfied researchers worked in centres that had strong leadership and support, collegial relationships among researchers that enhanced their working environment by fostering collaboration and sharing of equipment and other resources, and facilitating mentoring of young investigators [25].

The inadequate salaries noted by Argentine researcher can also be seen elsewhere. A survey of members of the American Society of Cell Biology saw only 39% of survey respondents indicate satisfaction with their financial compensation [26]. In Australia, both funding and infrastructure support are also researchers’ greatest concerns [27].

### Facilitators and Barriers to the Use of Health Research in Health Policymaking

The 15 facilitators and barriers to research use which we asked about were chosen from the literature as well as from the semi-structured in-depth interviews conducted previous to the survey design and implementation. At the time this research was undertaken, we were unable to identify any surveys on LMIC health researchers’ perspectives of facilitators and barriers to research use and impact on policymaking to date, therefore, we drew from studies based on the policymakers’ perspectives [28–31]. This research shows that there are both differences and similarities in policymakers’ and researchers’ perspectives.

Similar to findings from Lavis et al. (2005), we found that researchers perceived relationships between researchers and policymakers to be strong facilitators for research use in policymaking [32]. This finding is also reflected in a recently updated systematic review on barriers to and facilitators of the use of evidence by policymakers, where the most frequently reported facilitators were collaboration between researchers and policymakers, and improved relationships and skills [4]. The researchers we surveyed also clearly indicated that there was a lack of interaction between researchers and decision-makers and that researchers’ relationships with decision-makers should be based on merit and scientific credibility, not political connection. In a later study, Lavis et al. (2010) found that the existence of structures and processes to link researchers and their target audiences was a significant predictor of researchers establishing or maintaining long-term partnerships [11]. A recent evaluation of the Canadian Institutes of Health Research (CIHR) knowledge translation funding program identified the existence of a meaningful partnership between researchers and knowledge users as a catalyst for increasing both the relevance of research and the use of research[33, 34]. The concept of a meaningful partnership is expanded upon in detail in the CIHR evaluation, however it is worth noting that it matches closely to several factors identified as important in this study, such as ‘trust.’

### Perceptions and Attitudes of Researchers towards Decision Makers and Policymaker

Despite Argentina’s long scientific tradition, there are few links between research results and policy. At the core of the issue is the disconnect between political decision-making and the health research system. This disconnect between systems in Argentina, coupled with weakened technical capacity on the part of decision-makers and the perception of fewer research resources available, underscores the difficulty in supporting health policymaking with research evidence in Argentina.

There is no single optimal organisational structure to facilitate evidence-based health policymaking, mainly due to the fluidity of policy goals and interaction of a complexity of policy-influencing factors. Use of evidence within an organisation and at the institutional level largely depends on the policymaking context and culture of the units where policies are developed and varies depending on the issue and the type of scientific advice being sought (e.g., advisory, operational or regulatory), as well as the availability of the evidence [35–39].

Researchers’ indications that Argentine decision makers are only “a little” aware of the most urgent health problems in the country reflects a perceived strong disconnect between policymakers and the health needs of the Argentine population. This is reinforced by researchers’ perception that policymakers are highly motivated by self-interest rather than in fulfilling their public service role. In an interesting parallel, von Lengerke et al.’s (2003) survey of 719 policymakers in six high-income European countries found that political will was one of the most crucial determinants of policy impact [40]. The study authors show that research utilization may partially compensate for a lack of political will; this may present potential opportunities for research to play a role in contributing to policymaking in instances of political apathy, ambivalence, or in situations where information is required to drive decision-making.

Researchers surveyed in this study were divided as to how involved they would like to be in the policymaking process. Whereas 81.1% had never been involved in health policymaking or contributed to decision-making, approximately 90% had indicated that they would like to be involved. This shows that Argentine researchers are willing to being consulted and to contribute their expertise to inform decision-making, despite only 19% having ever worked with policymakers. With the data collected by this survey, it is difficult to determine the behavioural, cultural, or structural/institutional reasons for the disconnect between researchers’ desires to be involved and actual involvement.

### Possible Strategies for Connecting Research to Policymaking

Results from studies included in Orton et al.’s (2011) systematic review on the use of research evidence in public health decision-making processes suggest that in order to increase the use of research evidence in public health policy, strategies are required to encourage two-way communication between researchers and decision-makers; the environment within which decision makers work, in terms of structure and rewards, should be adapted to encourage the use of research evidence; decision-makers need training to increase their ability to access and interpret research outputs; and researchers require training and support to increase their ability to produce evidence of use to policy makers, to clearly present the main findings, and to effectively disseminate them to the relevant audience [39].

Improving personal contact and the building of relationships between researchers and policymakers in Argentina will require taking into account the perceptions of policymakers, as highlighted by researchers in this study. One potentially important consideration in establishing and growing relationships between researchers and policymakers, and improving research use in Argentine policymaking, is establishing trust [41–43]. Moorman, Zaltzman and Deshpande (1993) investigated the role of trust between knowledge users and knowledge providers (researchers) [44]. They suggest that a user’s trust in a researcher affects the user’s relationship processes with the researcher, including the quality of interactions, the extent to which the researcher is involved in the research process, and the user’s commitment to the specific research relationship. Trust increases research utilization because it facilitates higher quality interactions between users and researchers, higher levels of researcher participation and greater levels of user commitment to the relationship [44].

Different interventions exist for improving personal contact between researchers and policymakers, as well as for improving access to and information about research results. For example, in the Netherlands, researcher-policymaker collaborations are indirectly mandated by the Dutch government through a law encouraging evidence-based policy development by requiring that local health policy be based on epidemiological research [45]. In addition, research funders can design funding arrangements to incorporate knowledge users (e.g. policymakers amongst others), into research grants, which has been shown to more likely influence the behaviour of knowledge user partners, and lead to the creation of real world applications [33].

In addition to increasing a policymaker’s awareness of research and research processes, donors and research funders can also help facilitate institutional arrangements that link research activities with the political level so that the researchers are aware of the state-level priorities. This may include funded research sabbaticals within policy development units for new and established researchers or opportunities for collaboration between universities and student research, and policy development units. In Canada, the Canadian Institutes of Health Research (CIHR) have funded a successful ‘Partnerships for Health System Improvement (PHSI)’ program, which supports teams of researchers and decision-makers interested in conducting applied and policy-relevant health systems and services research. CIHR has found that research is more likely to be used in policy and practice when researchers work hand-in-hand with decision makers [46]. In addition, the Global Health Research Initiative, a Canadian government partnership between CIHR, the Department of Foreign Affairs, Trade and Development, and the International Development Research Centre, through various research programs, has encouraged Canadian and LMIC researchers to partner, design research programs so that LMIC researchers and decision-makers are required to be co-principle investigators on research projects.

Approximately 48.1% of Argentine researchers surveyed indicated that research being conducted ‘by a research leader’ was the most influential determinant of its eventual uptake into policy. One might believe that a research leader might be more trusted by a policymaker than a less experienced investigator. In a study on how policymakers find and assess public health researchers, it was found that policymakers valued researchers who had credibility across the three attributes seen as contributing to trustworthiness: competence (an exemplary academic reputation complemented by pragmatism, understanding of government processes, and effective collaboration and communication skills); integrity (independence, “authenticity”, and faithful reporting of research); and benevolence (commitment to the policy reform agenda)[43]. To help policymakers identify and access researchers, researchers and research leaders might be connected to policymakers using intermediary tools, such as a researcher database. A database which maps researchers and their areas of expertise, that is easily accessed by policymakers, like a professional directory, could be a useful and successful intervention. Existing government research institutions that fund the health researchers (like CONICET) would be well-positioned to take the initiative in this venture, given the high caliber of scientists it funds and familiarity with their work. Research leaders ought to be identified by experts, highlighted in this database, and connected to health policymakers, either when a specific policy is being developed to act as a content expert throughout the process or to serve as an external expert and reference as needed. This contact would ideally be initiated by the policy decision-maker, however a ‘knowledge broker’ entity, may also be useful.

Knowledge brokers are individuals or organizations acting as knowledge translation platforms; these can take the shape of ‘intermediary organizations, such as regional networks, dedicated institutional mechanisms and funding organizations’ [47]. Knowledge broker roles and related concepts have emerged as a new stream of research as facilitators for evidence-informed policymaking[4]. Critical factors for the success of a knowledge brokering mechanism are that the process is carefully designed and structured to bring together the scientific research community and policymakers, and that there is some institutional embedding of the process, taking the form of an intermediary organization [47–49]. In Argentina’s case, independent third parties outside of government and academia, such as think tanks or health policy analysis institutes [50], may find the most success.

A longer-term option would be to establish technical analysis units within a formalized institutional structure, where trained knowledge brokers are easily accessible to policymakers. A clearly defined knowledge transfer process should be implemented. This should be spearheaded by decision-makers, directors and team leaders, and adapted to the culture of the policymaking group or department. Researchers should be part of the knowledge transfer strategy consultation process, and professional facilitators should be hired to mediate the strategic planning process. Various knowledge translation self-assessment tools currently exist for research organizations to identify gaps in capacity and infrastructure of knowledge translation support at the organizational level, and which may be used to create supportive knowledge translation infrastructure, and facilitate interactions between researchers and target audiences to exchange questions and research findings [35, 51].

Ultimately, for evidence-based policymaking to work, an organizational-level mandate requiring that policy development and decision-making be based on evidence may contribute to the highest likelihood that policies be based on research evidence. For this vision to be realized, it will also be important that a leader be identified to champion the adoption of such a mandate, and to promote an evidence-based policymaking approach within the Ministry of Health, and other ministries. Individual- and organisational-level factors are the critical factors to target in the design of interventions to increase research use in a public health policy context, with specific attention paid to the relevance of the research and skills for research use [52].

### Limitations

We attempted to profile the researchers based on their desire to be involved in the policymaking process and the frequency of the researchers’ involvement with and contribution to decision-making. However, this was methodologically problematic because of the survey design, data structure and the small sample size resulting in diminished statistical power. Due to the lack of diversity of respondents, we were limited in gaining more meaningful information beyond the results we already had. Grouping the categories to try to increase sample size in the categories (e.g. public health/epidemiology/environmental health, compared to clinical medicine, compared to lab sciences) did not provide additional information of note.

Fewer than 10% of Argentine researchers surveyed had less than 10 years experience, meaning that our respondents were mostly mid- to senior-level researchers, with almost half having more than 20 years research experience. As such, we do not have the perspectives of less experienced researchers. Approximately 80% of our respondents had doctoral degrees and comprised mainly researchers in the biomedical sciences. Recognizing that this may not be representative of the Argentine research community, we acknowledge that these factors are likely due to the predominant population of CONICET researchers in our sample and the stringent CONICET standards for funding the highest calibre scientists. In addition, we did not ask researchers their age, as we believed that asking about their years of research experience was more relevant to the objectives of this study. However, we acknowledge that there may be generational and experiential perspectives that may be related to the age of the researcher, and that we may have overlooked these in this study.

Despite attempts to include as many as possible, we may have missed a significant segment of public health researchers. The list of researchers provided to us reflects a research profile typical of Argentina’s historical focus on funding research in the biomedical sciences. Although this was the most information we had on the health researcher population in Argentina, we would caution interpretation that this group of respondents is truly representative of all Argentine health researchers. Furthermore, it is probable that laboratory-based and clinical research has less direct relevance to policymakers. Accordingly, it is possible that this may limit researcher and decision-maker interactions, although this does not invalidate the researchers’ perceptions presented in this study.

Linguistic or cultural differences may have affected respondents’ interpretation of select questions. We tried to address this by conducting the qualitative phase of the research first, as well as pilot-testing the survey questionnaire several times. Researchers’ lack of familiarity with the terminology and concepts linked to knowledge translation may also have affected responses.

We were limited by having to keep the survey relatively short, given that it was self-administered and that no incentives were offered. Questions were edited for brevity and conciseness, which resulted in having to find a balance between breadth and depth of the questions. For example, we asked about the researchers’ publication productivity (a quantity) as a proxy for research productivity, and did not track the mean impact factor of the published articles (an index of quality).

## Conclusion

Improved understanding of policymakers and the policymaking environment by researchers, as well as an enhanced understanding of the value of evidence-based decision making, could lead to significant health systems benefits. These benefits might include more effective use of intellectual resources, better return on investments in health research via improved health outcomes, and/or more efficient and accountable use of public revenue within publicly-administered institutions. Accordingly, this research aimed to build a foundation for the development of stronger national health research systems, and improved linkages between research and policymaking. Although the results of this study are specific to Argentina, some may be applicable to other low- and middle-income country contexts, as well as high-income country contexts. Several of the findings uncovered in our research are consistent with findings of other research in the field from both developing and developed country contexts. For example, issues of ‘trust’ amongst researchers and research-users as a determinant of the success of research to action processes have been identified in our research, but also in research from Canada.

With health systems and national health research systems gaining greater prominence on the global stage, countries will be held ever more accountable to their constituencies for the decisions made in national health programming. Developing countries will be under scrutiny not only from their constituents but also from their donors. As we move into the post-2015 development era, there will also be greater pressure to demonstrate the effectiveness and results of interventions that improve health outcomes. In this respect, in-country health researchers will have a larger role to play in helping set national research priorities and in linking with decision-makers.

## Supporting Information

S1 File Questions Adapted from the WHO HRSA Initiative. (DOCX)

S2 File Web Survey Response Rate Improvement Efforts. (DOCX)
