# Supplementary material for: Survey of Argentine Health Researchers on the Use of Evidence in Policymaking
Source: PLoS One. 2015 Apr 30;10(4):e0125711. doi: 10.1371/journal.pone.0125711 (PMC4415923; doi:10.1371/journal.pone.0125711)
Supplement: S1 File — (DOCX) [file pone.0125711.s001.docx]

# S1 Questions adapted from the WHO’s HRSA Initiative

The World Health Organization’s Health Research Systems Analysis (HRSA) Initiative^[[1]](#footnote-1)^ has worked to describe, analyze and strengthen capacity of national health research systems in 13 low- and middle-income member countries, which include Brazil, Cameroon, Costa Rica, Indonesia, Islamic Republic of Iran, Kazakhstan, Lao PDR, Malaysia, Pakistan, Russian Federation, Senegal, Tanzania, and Thailand. The HRSA Initiative has made an effort to develop a core set of 14 indicators and 42 key descriptive variables through a collective benchmarking process. Recognizing that for this online survey we needed to balance the thoroughness of questions asked with survey length in order to optimize response rates (longer surveys have lower response rates), HRSA indicators were chosen selectively and adapted.

For example, in an attempt to gauge how happy/satisfied researchers were and how supportive the research environment is for those surveyed, we included an adapted version of the HRSA domain where we asked health researchers to give a rating between 0 and 100, where 100 represented optimal conditions or an ideal situation. The following domains were rated by researchers:

- Range and breadth of health researcher networks they were a part of and had access to
- Transparency of the funding process
- Quality of the work space and facilities
- Encouragement of collaboration with others
- Opportunities to present, discuss and publish results
- Relevance of health research activities to health problems
- Education and continuous training
- Nurturing of careers
- Access to information

We also asked how many submissions had been made to ethics committees for ethical approval of research. This question also came from WHO’s HRSA survey into health research systems and is meant to more accurately gauge levels of activity in human subjects research.

The questions included in our survey were:

How do you most commonly share your research findings, or how do you plan on disseminating your work? Please indicate the option you use the most.

- I do not disseminate my findings
- Publications in journals, books, reports, etc.
- Presentations at conferences (posters, plenary sessions)
- Web listserves

What is the total number of health research publications that you have produced in 2005-2009 according to:

| A. Articles published in peer-reviewed journals, in Spanish |  |
| --- | --- |
| B. Articles published in peer-reviewed journals, in English |  |
| C. Published books |  |
| D. Written/published reports |  |
| E. Working papers |  |
| F. Systematic reviews (e.g. Cochrane reviews or others) |  |

We also adapted HRSA’s indicator “Proportion of health research institutions with access to both national and international health journals (print or electronic versions), during 1997-2001” to determine whether the researchers had access to both national and international health journals (print or electronic versions).

1. More information about this Initiative can be found at their website: http://www.who.int/rpc/health_research/en/. [↑](#footnote-ref-1)
