# Supplementary material for: Survey of Argentine Health Researchers on the Use of Evidence in Policymaking
Source: PLoS One. 2015 Apr 30;10(4):e0125711. doi: 10.1371/journal.pone.0125711 (PMC4415923; doi:10.1371/journal.pone.0125711)
Supplement: S2 File — (DOCX) [file pone.0125711.s002.docx]

# S2 Web Survey Response Rate Improvement Efforts

Our survey was an interactive web survey comprising 28 web pages, where each page had one to three questions, and was designed to enhance the ease and flow of moving through the survey. Some pages had questions that required more introspection by the respondent, but these were deliberately followed by shorter questions not requiring much introspection (e.g. type of organization researcher works for).

This software used permitted control of skips and branching and embedded edit checks. The program was able to include progress indicators and was flexible in allowing colour, question and page formatting. Multimedia design elements and layout were important considerations when creating the survey, with special attention being paid to graphics, color, typography, font, size and spacing. In the absence of an interviewer to motivate the respondent or to provide guidance on how to answer each question, respondents will seek such information from the instrument itself, using both the verbal and visual elements of the interface (Ware, 2000).

When respondents clicked on the embedded link in the emailed invitation, a Web page opened introducing them to the survey. A unique identifier was created for that survey by the emailed link. Once the participant began the survey, he or she was able to return to complete the survey by clicking on the link provided in the e-mail, without being double-counted. Upon survey completion and submission, the respondent was not able to modify or change answers.

Efforts to improve response rate included:

- Creation of a separate Johns Hopkins Bloomberg School of Public Health (JHSPH) email account in the name of our Argentine collaborator, a senior scientist with the National Research Council and a very well-known and respected scientist in Argentina. This was done to improve response rates based on potential familiarity of a known person as well as to avoid spam filters.
- Enhanced credibility via university sponsorship again apparent by inclusion of JHSPH logo in the online survey.
- Emails were initially sent in batches of 10-12 email addresses to avoid SPAM filters. Subsequent email reminders were sent in batches of 8-9 email addresses.
- Inclusion of a progress bar at the bottom; unlike in scrollable survey forms or mail surveys, interactive Web surveys are able to incorporate this.
- Inclusion of motivational messages at one-third and two-thirds completion (as suggested by Couper et al. (2001)).
- Formatting the survey for easier readability.
- Mixing long and short questions to avoid tedium.
- Using Argentine national colours of powder blue and white throughout the survey.
- Limiting the number of questions for which an answer was mandatory to continue, in an effort to reduce item non-response.
- Grouping related items to reduce the time taken to complete the survey and reduce respondent effort to re-orient to new question and response formats (as per Couper et al. 2001).
- Using radio buttons and option boxes as much as possible, as per Couper et al.’s assumption that clicking a radio button requires less effort on the part of respondents than typing a response in a box and that a mouse can be used for all input, rather than the keyboard. Furthermore, radio buttons restrict the range of permissible answers, thereby preventing out-of-range answers.
- Designing the questions so as to be visually facilitating; for example, when respondents were required to enter a numerical value, the question was designed to be visually facilitating by entering these in a vertical format, as is the cultural norm for adding up a set of numbers (Couper et al. 2001).
- An edit check for numerical responses within a certain range was built-in. In these cases, a shorter entry text box was used; Couper et al. (2001) found that short entry boxes had a greater percentage of respondents completing the task than the long entry box version, again suggesting that the longer box encouraged respondents to provide more information than was required.
- Grouping of items in multi-item questions took into account the type of items being considered and their relationship to each other.

Seasonality was also taken into account, recognizing that the months in which the survey was implemented (May 2010 to June 2010), were the months that Argentines were the least likely to take holiday and be away from their computers. Furthermore, it was also the time period where reports were due to CONICET regarding researcher’s research activities throughout the year.

# 
